# An apicoplast-localized deubiquitinase contributes to the cell growth and apicoplast homeostasis of *Toxoplasma gondii*

**DOI:** 10.1186/s13567-023-01261-y

**Published:** 2024-01-17

**Authors:** Jie Xia, Yimin Yang, Xueqiu Chen, Kaiyue Song, Guangxu Ma, Yi Yang, Chaoqun Yao, Aifang Du

**Affiliations:** 1 Institute of Preventive Veterinary Medicine and Zhejiang Provincial Key Laboratory of Preventive Veterinary Medicine, College of Animal Sciences, Zhejiang University, Hangzhou, 310058 Zhejiang China; 2 Department of Biomedical Sciences and One Health Center for Zoonoses and Tropical Veterinary Medicine, Ross University School of Veterinary Medicine, P.O. Box 334, Basseterre, Saint Kitts and Nevis

**Keywords:** *Tg*OTU7, *Toxoplasma gondii*, apicoplast, deubiquitinase, ubiquitination

## Abstract

*Toxoplasma gondii* is among the most important parasites worldwide. The apicoplast is a unique organelle shared by all Apicomplexan protozoa. Increasing lines of evidence suggest that the apicoplast possesses its own ubiquitination system. Deubiquitination is a crucial step executed by deubiquitinase (DUB) during protein ubiquitination. While multiple components of ubiquitination have been identified in *T. gondii*, the deubiquitinases involved remain unknown. The aim of the current study was to delineate the localization of *Tg*OTU7 and elucidate its functions. *Tg*OTU7 was specifically localized at the apicoplast, and its expression was largely regulated during the cell cycle. Additionally, *Tg*OTU7 efficiently breaks down ubiquitin chains, exhibits linkage-nonspecific deubiquitinating activity and is critical for the lytic cycle and apicoplast biogenesis, similar to the transcription of the apicoplast genome and the nuclear genes encoding apicoplast-targeted proteins. Taken together, the results indicate that the newly described deubiquitinase *Tg*OTU7 specifically localizes to the apicoplast and affects the cell growth and apicoplast homeostasis of *T. gondii*.

**Supplementary Information:**

The online version contains supplementary material available at 10.1186/s13567-023-01261-y.

## Introduction

*Toxoplasma gondii* is a parasitic protozoan that infects almost all warm-blooded creatures, including humans [1]. Although most infected people do not show clinical signs or symptoms, individuals with compromised immunity, such as those with HIV/AIDS or who have undergone organ transplantation, may suffer from severe clinical consequences [2, 3]. Furthermore, pregnant women can pass the parasite to their foetuses through the placenta, possibly leading to severe congenital defects or even abortion/stillbirth. The same clinical outcomes might also occur for domestic animals infected with *T. gondii*, resulting in enormous economic losses worldwide [4, 5]. Effective chemotherapy, control and prevention are essential for minimizing the effects of *T. gondii* on human and animal health as well as reducing economic losses.

New drugs are greatly needed in the war against *T. gondii* worldwide. Primary targets for chemotherapy are usually the biochemical pathways unique to the parasite. The apicoplast of *T. gondii* has attracted increased amounts of attention. It is a plastid organelle containing a circular double-stranded DNA genome of approximately 35 kb that is surrounded by four plasma membranes [6]. This organelle is essential for parasite viability due to its participation in various biosynthetic pathways, such as fatty acid [7] and haem [8] biosynthesis pathways. Interruption of apicoplast-associated metabolism in *T. gondii* usually results in a loss of ability to infect host cells [9] and even death [10, 11]. Several lines of evidence suggest that the apicoplast originated from engulfed red algae and evolved through endosymbiosis during evolution [12, 13]. Because of this unique origin, the apicoplast possesses a set of metabolic pathways that vastly differ from those of mammalian cells. Therefore, the apicoplast is a promising new drug target for treating *T. gondii* infections.

Ubiquitination, a well-known post-translational modification (PTM), plays a pivotal role in the regulation of a wide range of biological processes, including protein degradation, signal transduction, and DNA repair [14–18]. PTM involves tightly controlled activation, conjugation, ligation, and deubiquitination, which are each carried out and regulated by specific enzymes [15, 19]. The ubiquitin system includes (1) all ubiquitinated proteins, (2) enzymes that catalyse the addition/removal of ubiquitin, and (3) proteins that bind to ubiquitinated proteins. The enzymes responsible for removing a ubiquitin motif from substrate proteins are called deubiquitylating proteases or deubiquitinase (DUBs) [20]. The *T. gondii* genome encodes the following different types of DUBs: ubiquitin-specific proteases (USPs), ubiquitin C-terminal hydrolases (UCHs), ovarian tumour proteases (OTUs), Josephins and metalloproteases [21]. However, compared with extensive studies on the ubiquitination system and associated components in *T. gondii*, little is known about DUBs in this parasite [22–25].

Most apicoplast proteins are encoded by the nuclear genome and imported although the organelle contains its own genome [24]. Translocation of these proteins requires the apicoplast’s own designated ubiquitination system [25]. The interference of ubiquitination by gene knockout partially blocks this process [25–27]. The endoplasmic reticulum-associated protein degradation (ERAD) network functions as a translocon across the second outermost membrane of the apicoplast in a ubiquitylation-dependent manner [28, 29]. Several apicoplast ERAD-associated ubiquitination components have been identified in *T. gondii* [25, 26], but none are deubiquitases. In *P. falciparum*, an OTU deubiquitinase localized to vesicular structures associated with the apicoplast is involved in apicoplast protein transport and homeostasis [27]. Members of the OTU family have been reported to be involved in the NF-κB signalling pathway [30], autoimmunity inhibition [31], and various other important pathways. In apicomplexan parasites, OTU DUBs are implicated in nondegradative cellular processes such as parasite development [32]. We recently identified an apicoplast-localized DUB belonging to the OTU family, *Tg*OTU7. The aim of the current study was to determine the distribution of this fungus and its role in *T. gondii infection*. We characterized its expression profile, examined its deubiquitinase activity by immunofluorescence and Western blot, and explored its pivotal roles in apicoplast homeostasis and apicoplast-related gene transcription using knockout mutants. Taken together, our data reveal the “ubiquitin code” in *T. gondii*, laying a solid foundation for identifying new drug targets.

## Materials and methods

### Parasite and cell culture

*T. gondii* RHΔ*ku80*Δ*hxgprt* (RHΔ*ku80*) and its mutants were maintained in a confluent monolayer of human foreskin fibroblasts (HFFs) as previously described [33]. To isolate the tachyzoites, the samples were passed through a French press [34] and 5-μm syringe filter (Millipore, Darmstadt, Germany).

### Bioinformatic analysis

The signal peptide (SP) and transit peptide (TP) of the DUBs were identified by SignalP–5.0 [35] and ChloroP–1.1 [36], respectively. The sequence of *Tg*OTU7 and its homologues in other organisms were obtained from NCBI [37] and VEuPathDB [38]. The sequence similarity of these homologues among different organisms was analysed with Geneious Prime (Dotmatics Geneious, New Zealand, USA). The conservation of amino acid residues in the active site was demonstrated by MUSCLE alignment, and a phylogenetic tree was constructed with MEGA X [39] using the neighbour-joining method with 1000 bootstrap replications. The catalytic motifs of both *Tg*OTU7 and human OTUs (HsOTUs) were predicted utilizing MEME [40].

### Epitope tagging

A homology-directed repair template and a CRISPR/Cas9 plasmid with a SgRNA sequence were prepared by introducing 3 × HA or 6 × HA tag at the 3’ terminus of the genes of interest. The template was amplified from the pLinker-BirA-3 × HA-HXGPRT-LoxP (86668; Addgene) or pLinker-6 × HA-HXGPRT-LoxP (86552; Addgene) vectors, which contained a homologous sequence 40 bp upstream of the stop codon and 40 bp downstream of the Cas9 cleavage site. The SgRNA sequence was designed online at EuPaGDT [41] and ligated to the pSAG1::Cas9-U6::*Bbs* I plasmid modified from pSAG1::CAS9-U6::sgUPRT (plasmid 54467, Addgene). The template and plasmid were simultaneously electroporated into 1 × 10^7^ RHΔ*Ku80* tachyzoites as previously described [33]. Positively transfected parasites were selected with mycophenolic acid (25 mg/mL) and xanthine (50 mg/mL), and individual clones were obtained by limiting dilution performed in a 96-well plate. The epitope tags were confirmed by PCR, IFA, and Western blotting.

### *Tg*OTU7 knockout and complementation

To construct *Tg*OTU7-KO plants, a modified CRISPR/Cas9 plasmid and a repair template were first prepared. The CRISPR/Cas9 plasmid was generated as described in the previous section. The repair template consisted of a DNA fragment containing the dihydrofolate reductase (DHFR) cassette selection marker flanked by 40 bp each at the specific 5’ and 3’ homology regions of *TgOTU7*. Electroporation of the RHΔ*Ku80* tachyzoites was performed as previously described [33]. *Tg*OTU7-KO plants were cultured in culture medium supplemented with 3 mM pyrimethamine, after which the results were confirmed via PCR.

To construct the *Tg*OTU7-KO-complemented strain (*Tg*OTU7-COM), the coding sequence of the *TgOTU7* gene was amplified and inserted into the pLinker-6 × HA-HXGPRT-LoxP upstream of the 6 × HA sequence. The repair template was a DNA fragment containing the *TgOTU7*-6 × HA-HXGPRT cassette flanked by 40 bp each at the specific 5’ and 3’ homology regions of *TgOTU7*. The CRISPR/Cas9 plasmid was constructed with an SgRNA that cut the DHFR sequence. Electroporation of the *Tg*OTU7-KO tachyzoites was performed. *Tg*OTU7-COM was selected in culture medium supplemented with mycophenolic acid (25 mg/mL) and xanthine (50 mg/mL) and confirmed by PCR.

### Immunofluorescence assay (IFA)

IFA was performed as previously described [42]. Briefly, HFFs were cultured on 14-mm-diameter glass coverslips until confluent in a 37 °C incubator supplied with 5% CO_2_. The cells were then infected with 1 × 10^5^ tachyzoites per coverslip and incubated for an additional 24 h. Afterwards, the cells were fixed in 4% formaldehyde for 15 min, permeabilized with 0.25% Triton X-100 for 30 min, and blocked with 1% bovine serum albumin (BSA) in phosphate-buffered saline (PBS) at 37 °C for 1 h. The primary antibodies used were rabbit monoclonal anti-HA tag (C29F4) (1:500) (Cell Signaling Technology, Boston, USA), mouse anti-*Tg* (1:2000), mouse anti-IMC1 (1:1000), mouse anti-CPN60 (1:1000), mouse anti-sortilin (1:1000), mouse anti-Centrin 1 (1:1000), mouse anti-ROP14 (1:1000), mouse anti-GRA16 (1:1000), rabbit anti-IMC1 (1:1000) and rabbit anti-GAP45 (1:1000). The antibodies were generated in the laboratory as previously described [43] unless a source was provided. The secondary antibodies used were goat anti-mouse IgG Alexa Fluor 488/594/647 (1:1000) and goat anti-rabbit IgG Alexa Fluor 488/594/647 (1:1000) (Invitrogen, Carlsbad, USA). The nuclear dye 4’,6-diamidino-2-phenylindole (DAPI) was used at a dilution of 1:2000. The cells were viewed under a Zeiss LSM 880 microscope, and images were processed with ZEN 2.3 software (Zeiss, Oberkochen, Germany).

### Western blotting

For immunoblotting, parasites or host cells were lysed in lysis buffer (25 mM Tris–HCl, 200 mM NaCl, 10 mM NaF, 1 mM Na_3_VO_4_, 25 mM beta-glycerol phosphatase, 1% NP-40) supplemented with a protease inhibitor cocktail (Bimake, Shanghai, China) for 30 min on ice. Total cellular lysates were separated via SDS‒PAGE, and proteins were transferred to polyvinylidene fluoride (PVDF) membranes (Millipore), which were subsequently probed with the appropriate primary antibodies and corresponding secondary antibodies conjugated to horseradish peroxidase (HRP). The PVDF membranes were then subjected to treatment with enhanced chemiluminescence (ECL) reagents (Fdbio Science, Hangzhou, China), and the signals were immediately detected with a ChemiDoc™ chemiluminescence system (Bio-Rad, Hercules, USA).

The primary antibodies used were mouse anti-FLAG (1:1000), rabbit anti-HA (1:1000) and mouse anti-β-tubulin (1:2000) (Cell Signaling Technology). The following HRP-conjugated secondary antibodies were used: goat anti-mouse (1:5000) (Fdbio Science) and goat anti-rabbit (1:5000) (Fdbio Science).

### Quantitative PCR analyses

Genomic DNA (gDNA) was extracted from purified *T. gondii* tachyzoites using a TIANamp Genomic DNA Kit (TIANGEN, Beijing, China) following the manufacturer’s instructions. Total RNA was extracted from purified *T. gondii* tachyzoites using TRIzol (Invitrogen), and the RNA was transcribed into cDNA using a ReverTra Ace qPCR RT Kit (Toyobo) per the manufacturer’s instructions. The quantitative real-time PCRs (qPCRs) included 20 ng of gDNA/cDNA, SYBR® Green Real-time PCR Master Mix (Toyobo, Osaka, Japan), and specific primers. All qPCRs were performed on a T100 Real-Time PCR System (Bio-Rad, California, USA). The relative mRNA and gDNA levels were calculated using the 2^−ΔCT^ method [44]. Each experiment was repeated three times independently with three replicates each.

### Plasmids

DNA fragments encoding *TgOTU7*-FLAG (full length), *TgOTU7*-FLAG (aa 1–142), *TgOTU7-*FLAG (aa 143–347), and *TgOTU7*-FLAG (aa 149–285), which were codon optimized for expression in human cells, were individually cloned and inserted into the pRK5 vector. All the full-length and truncated TgOTU7 sequences were amplified from cDNA as described above, and the Flag tag was fused to the 3’ region. pRK5-HA-Ubiquitin-WT (referred to as pRK5-HA-Ub) (plasmid 17608; Addgene), pRK5-HA-Ubiquitin-K33 (plasmid 17607; Addgene), pRK5-HA-Ubiquitin-K48 (plasmid 17605; Addgene), pRK5-HA-Ubiquitin-K63 (plasmid 17606; Addgene), and pRK5-HA-Ubiquitin-KO (plasmid 17603; Addgene) were obtained from Addgene. The PLK5-HA-Ubiquitin-K6, pRK5-HA-Ubiquitin-K11, pRK5-HA-Ubiquitin-K27, and pRK5-HA-Ubiquitin-K29 plasmids were generated from a plasmid of HA-tagged ubiquitin lacking lysine, pRK5-HA-Ubiquitin-KO, through point mutation.

### Deubiquitination assay

The transfection and cotransfection of HEK293T cells were performed with polyethyleneimine (PEI) [45]. PEI transfection reagent was prepared by diluting 3.5 μL of PEI in 50 μL of DMEM, after which the mixture was mixed with the plasmids and incubated for 20 min. To optimize the concentration, various amounts of pRK5-*TgOTU7*-FLAG and pRK5-HA-Ub were added. For catalytic experiments, pRK5 plasmids containing different regions of *TgOTU7* were added along with pRK5-HA-Ub. For linkage specificity, pRK5-*TgOTU7*-FLAG was coadded with pRK5-HA containing different linkage types of ubiquitin. A mixture of PEI and DNA was subsequently added to the HEK293T cells, which were then incubated for 6 h so that the PEI-DNA complexes were taken up by the cells. The cells were then washed, and fresh cell culture media was added. Afterwards, the cells were left to grow and express the DNA of interest for 18 h and were prepared for Western blot analysis using anti-FLAG and anti-HA antibodies, with β-tubulin serving as a reference.

### Plaque assay

A plaque formation assay was performed as previously described [33]. Briefly, tachyzoites were seeded into a confluent monolayer of HFF cells in a 6-well plate (200 tachyzoites per well), which was subsequently incubated for 7 days at 37 °C in 5% CO_2_. Afterwards, the cells were gently washed with PBS, fixed with precooled methanol for 20 min, stained with 0.2% crystal violet for 10 min, dried, and photographed. Photoshop CC 2018 (Adobe, California, USA) was used to quantify the number and area of the plaques.

### Attachment and invasion assay

Freshly purified tachyzoites were added to HFF monolayers grown on glass coverslips in a 24-well plate (5 × 10^6^ tachyzoites per well) and cultured for 30 min. The cells and parasites were processed as previously described [33]. For the invasion assay, at least 10 random images were captured for each sample at 630 × magnification to determine the number of parasites and host nuclei. Yellow (overlapping red and green) and green tachyzoites were identified as extracellular (attached) and intracellular (invaded), respectively. The relative efficiency of invasion was determined by the formula: intracellular parasites/(extracellular + intracellular parasites) × 100%. The data were acquired from three independent experiments with three technical replicates each.

### Replication assay

Freshly purified tachyzoites were added to confluent HFF monolayers grown on glass coverslips in a 24-well plate (1 × 10^5^ tachyzoites per well) and incubated for 3 h. Uninvaded parasites were removed by washing in DMEM. At 24 h post-infection, the infected cells were treated as described previously [33]. Afterwards, at least 10 random images were taken for each sample under a magnification of 630 × , and each image was assessed for the number of parasites per vacuole. Three experiments, each consisting of three replicates, were conducted independently.

### Egress assay

HFF cells on a coverslip were infected with tachyzoites as previously described [33]. The sections were then washed to remove uninvaded parasites. After the cells were further incubated in the medium 36 h, the cells were incubated in 500 μL of 3 μM calcium ionophore in PBS for 2 min to induce egress of the parasites. The cells, including parasites, on the coverslip were then fixed with 4% paraformaldehyde and blocked in 1% BSA-PBS. The sections were labelled with mouse anti-*Tg* antibody and goat anti-mouse Alexa Fluor 594 antibody. The cells were then permeabilized in 0.25% Triton X-100 and labelled with rabbit anti-IMC1 and goat anti-rabbit Alexa Fluor 647. Finally, the nuclei were stained with DAPI. The percentage of egress was calculated by dividing the number of lysed vacuoles by the total number of vacuoles in a sample. Three independent experiments with three replicates each were conducted.

### Statistical analyses

The data from the plaque, invasion, replication, or egress assays are presented as the mean ± S.D. An unpaired t test or two-way ANOVA was performed for statistical analyses using GraphPad Prism 9.0 (GraphPad Software, Boston, USA).

## Results

### The deubiquitinase OTUB1 is not specifically localized at the apicoplast

**Figure 1:**
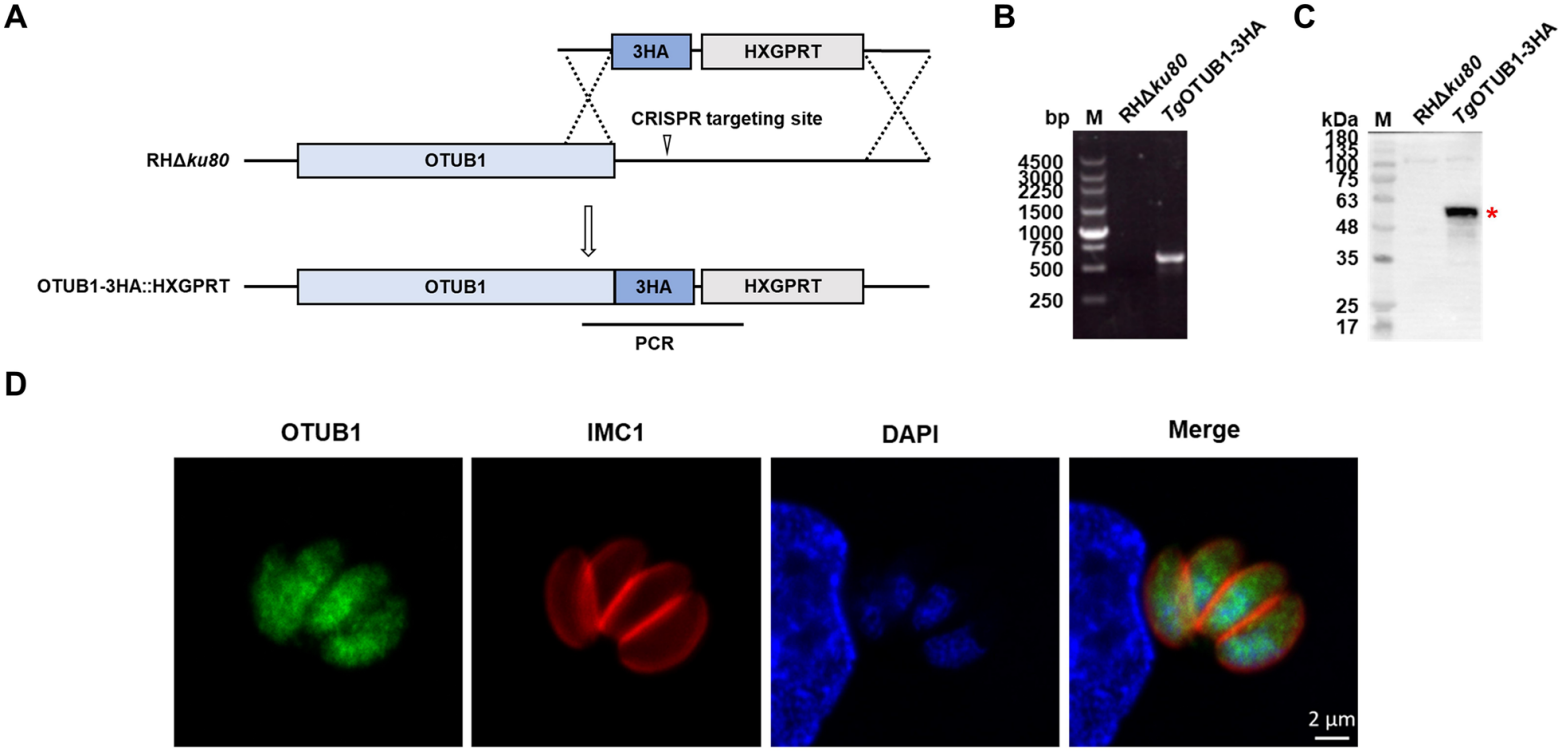
***Tg*OTUB1 is not specifically localized at the apicoplast.** **A** Schematic representation of the CRISPR/Cas9 strategy used to construct the endogenous *Tg*OTUB1-3HA-tagged tachyzoites. **B** PCR analysis of the 3 × HA-tag insertion at the 3′ end of the *TgOTUB1* gene. PCR targets are shown in Panel **A**. **C** The *Tg*OTUB1-3HA protein was detected via Western blotting using rabbit anti-HA antibodies. The expected size of the *Tg*OTUB1-3HA proteins is highlighted by a red star. **D** Immunofluorescence staining revealed the intracellular localization of endogenously tagged *Tg*OTUB1. Parasites were probed with rabbit anti-HA (green) and mouse anti-IMC1 (red, inner membrane complex 1). IMC1 was used to outline the tachyzoites, and DAPI (blue) was used as a nuclear stain.

Many ubiquitination-related proteins*,* such as a ubiquitin conjugating enzyme (E2_AP_) [25] and a plastid ubiquitin-like protein (PUBL), have been found in the apicoplast of *T. gondii* [26]. *Tg*OTUB1, a deubiquitinase of the OTU family, is predicted to target the apicoplast [25]. To confirm this, we tagged endogenous *Tg*OTUB1 with 3 × HA at the C-terminus (Figure 1A). A product of the predicted size for *Tg*OTUB1-3HA was obtained via PCR (Figure 1B). Furthermore, after Western blot analysis using anti-HA antibodies, proteins of the expected size were detected (Figure 1C). IFA revealed that *Tg*OTUB1 was widely distributed throughout the entire tachyzoite and exhibited puncta, including in the cytoplasm and nucleus (Figure 1D). The data clearly showed that *Tg*OTUB1 did not specifically target the apicoplast.

### The deubiquitinase OTU7 is expressed at the apicoplast in a cell cycle-dependent manner

**Figure 2:**
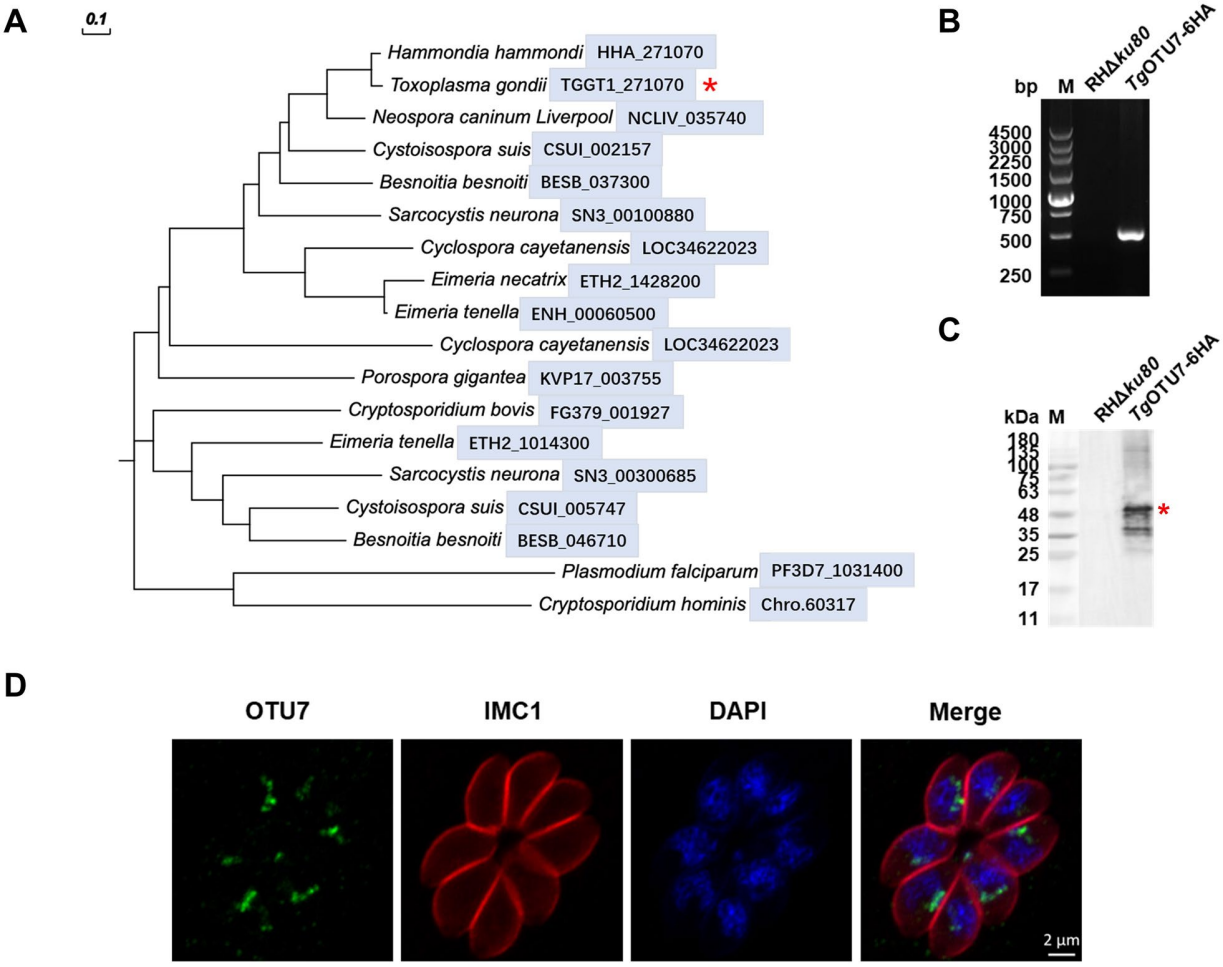
**Phylogenetic analysis, gene tagging identification and intracellular localization of *Tg*OTU7.** **A** Phylogenetic tree of OTU7 of different Apicomplexa species. A phylogenetic tree was constructed based on the amino acid sequences with the neighbour-joining algorithm using MEGA X. Bootstrap analysis was performed with 1000 replicates. The red star indicates OTU7 of *T. gondii*. The scale bar indicates the evolutionary distance between the sequences. **B** PCR analysis of 6 × HA-tag insertions at the 3′ end of the *TgOTU7* gene. **C** Western blot identification of the *Tg*OTU7-6HA protein using rabbit anti-HA antibodies. The expected size of the *Tg*OTU7-6HA proteins is highlighted by a red star. The bands of smaller size may be due to processed or degraded forms of *Tg*OTU7-6HA. **D** Immunofluorescence assay revealing the intracellular localization of endogenously tagged *Tg*OTU7. Parasites were stained with rabbit anti-HA (green), mouse anti-IMC1 (red) and DAPI (blue).

We next explored whether there is an additional apicoplast-targeting deubiquitinase in *T. gondii*. Most proteins targeting the apicoplast usually possess an SP and a TP [46]. Based on these findings, we identified a candidate, TGGT1_271070, which was annotated as a hypothetical protein in the ToxoDB [47]. However, the candidate was also designated as *Tg*OTU7 [22]. This predicted protein has a relatively close evolutionary relationship with the *Hs*OTUB1 and *Hs*OTUD6A clades (Additional file 1). A phylogenetic analysis revealed that *Tg*OTU7 has many homologues in apicomplexan parasites (Figure 2A). *Tg*OTU7 is phylogenetically rather distant from *Pf*OTU (PF3D7_1031400), the OTU targeted to the apicoplast of *P. falciparum* [27] (Figure 2A). The sequence homology between *Tg*OTU7 and *Pf*OTU was also low, at only 6.9% (Additional file 2).

**Figure 3:**
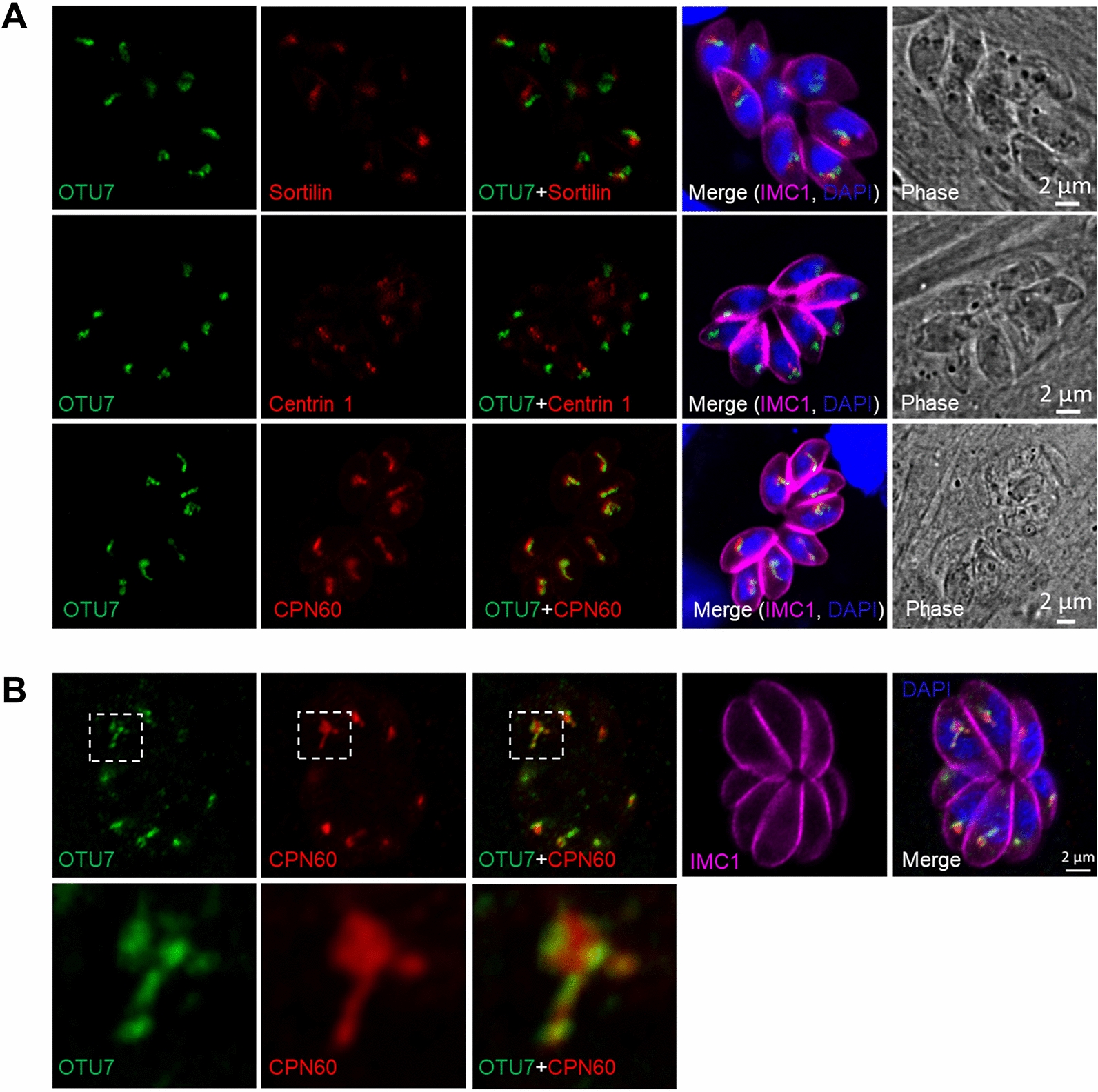
***Tg*OTU7 is localized at the apicoplast.** **A** Immunofluorescence colocalization analysis of *Tg*OTU7 (green) with Sortilin (red, Golgi apparatus, top panel), Centrin 1 (red, centrosome, central panel) and CPN60 (red, apicoplast, bottom panel). Parasites were also stained with rabbit anti-IMC1 and DAPI. **B** Colocalization of *Tg*OTU7 with CPN60. *Tg*OTU7-6HA (green); CPN60 (red).

We next ascertained the cellular localization of *Tg*OTU7 in *Toxoplasma*. We first tagged the endogenous gene with 6 × HA added at the 3′ end. Successful tagging was confirmed by PCR and Western blotting, as shown in Figures 2B and C, respectively. The expected size of the tagged *Tg*OTU7-6HA protein was 50 kDa, as indicated by the “*” in Figure 2C. Three additional proteins of 48, 37, and 35 kDa were also observed, possibly including processed or degraded *Tg*OTU7-6HA. *Tg*OTU7 was localized to the perinuclear region, as revealed by IFA (Figure 2D). To further determine the localization of *Tg*OTU7, various well-defined markers, including CPN60 for apicoplasts [48], Centrin 1 for centrosomes [49], and Sortilin for the Golgi [50], were used in colocalization analysis experiments. *Tg*OTU7 was clearly not localized at the centrosome or at the Golgi. However, our results indicated that this gene was located at the apicoplast, as shown by its partial colocalization with CPN60 (Figure 3A). Furthermore, *Tg*OTU7 seemed to surround the luminal protein CPN60, which suggested that *Tg*OTU7 resides in the periphery, probably the membrane of the apicoplast (Figure 3B).

**Figure 4:**
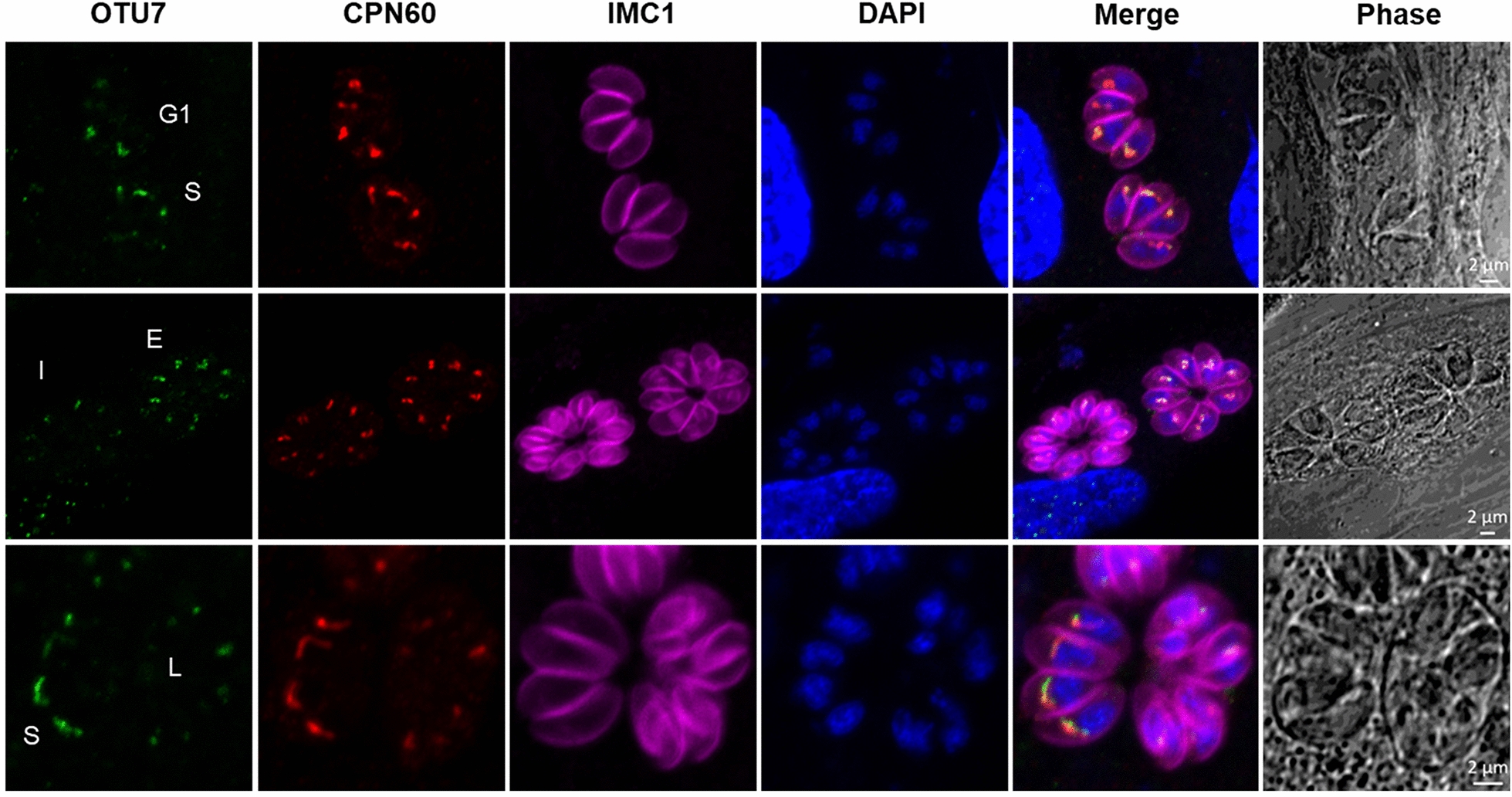
***Tg*OTU7 expression dynamics and localization among different cell cycle stages.** Parasites were stained with rabbit anti-HA (green, indicating *Tg*OTU7), mouse anti-CPN60 (red, an apicoplast luminal protein), rabbit anti-IMC1 (fuchsia, inner membrane complex 1), or DAPI (blue, nucleus). The G1, S, early (E), intermediate (I), and late (L) stages of cytokinesis were defined by the size, morphology and DNA intensity of the cell nucleus integrated with the extent of IMC1 labelling of the daughter scaffolds and CPN60 labelling of the apicoplast.

Tachyzoites of *T. gondii* undergo endodyogeny during asexual reproduction [51], which separates into cell cycle phases according to the progress of DNA replication and cytokinesis [52]; almost one-third of the *T. gondii* transcriptome is intricately regulated in each phase [53]. We next determined the localization of *Tg*OTU7 in relation to the cell cycle. The G1, S, and M phases are determined by nuclear size and shape as well as DNA content [22, 52]. The early (E), intermediate (I), and late (L) stages of cytokinesis were determined by the staining pattern of IMC1 [22]. At different cell cycle stages, *Tg*OTU7 was shown to colocalize with the apicoplast marker CPN60 by yellow fluorescence in IFA, which unequivocally confirmed that *Tg*OTU7 was localized to the apicoplast (Figure 4). Furthermore, *Tg*OTU7 was more abundant in the G1/S phase of DNA synthesis than in the E/I/L phase of cytokinesis (Figure 4). The log_2_ values (RMA-normalized signal intensity values of the *Tg*OTU7 gene) were used to analyse the mRNA levels, which corroborated the IFA results (Additional file 3). Taken together, these findings indicate that the expression of *Tg*OTU7 is intricately regulated during the entire endodogeny process at the apicoplast.

### The linkage-nonspecific deubiquitinating activity of *Tg*OTU7 is dependent upon the catalytic OTU domain

**Figure 5:**
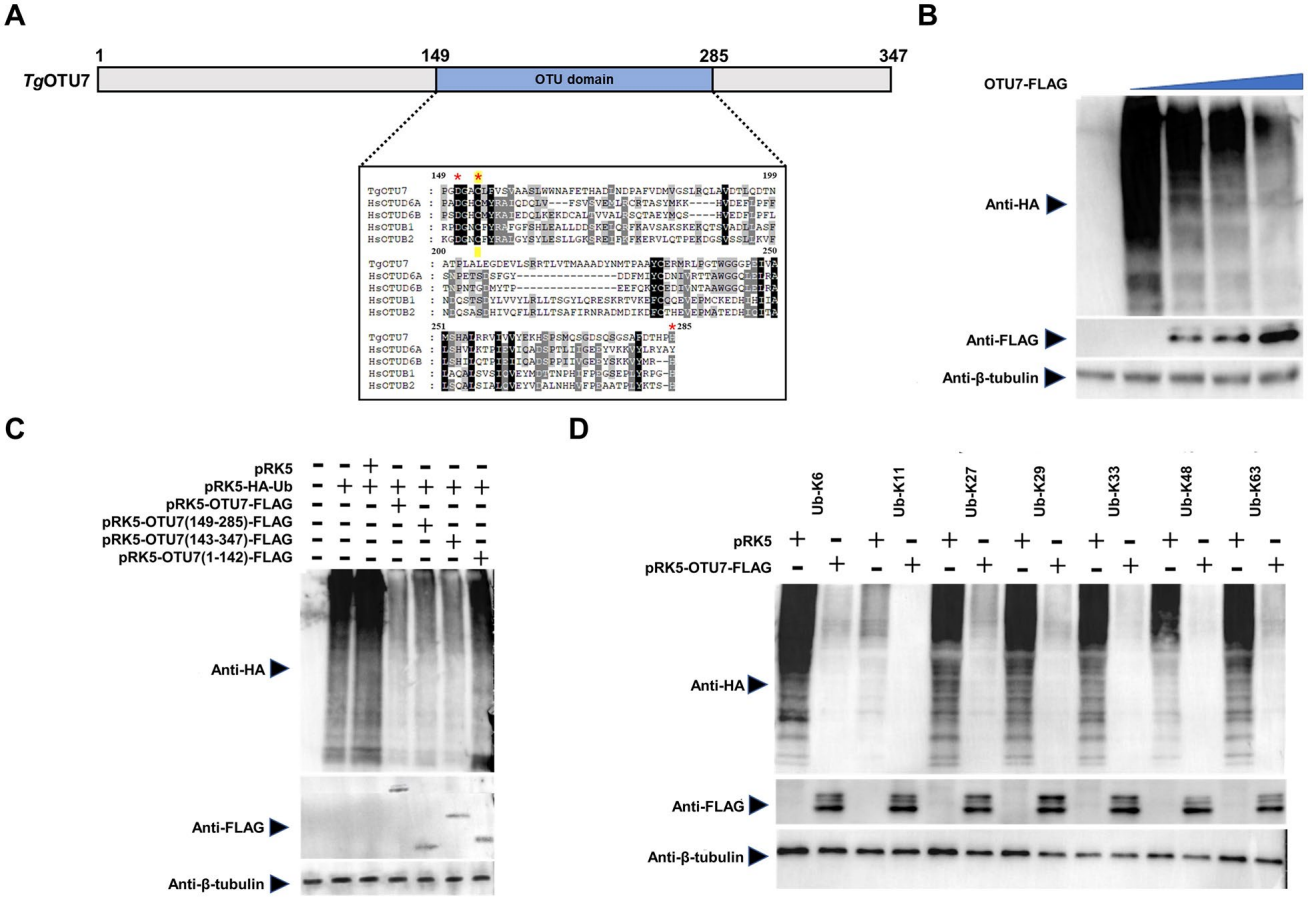
***Tg*OTU7 is a deubiquitinase.** **A** Schematic structure of *Tg*OTU7, highlighting the OTU domain. The sequences of the amino acid (aa) sequences of *Tg*OTU7 (TGGT1_271070), *Hs*OTUD6A (NP_056022.1), *Hs*OTUB1 (NP_060140.2), *Hs*OTUD6B (NP_057107.4), and *Hs*OTUB2 (NM_023112.4) are presented. Critical aspartate, cysteine, and histidine residues forming the catalytic triad are highlighted with red stars. **B** Deubiquitinase activity of *Tg*OTU7. The ubiquitination level of exogenous ubiquitin proteins was measured by Western blotting using individual antibodies against different antigens. Cellular lysates of HEK293T cells transfected with the pRK5-HA-Ub and pRK5-*TgOTU7*-FLAG plasmids were used. The anti-HA band indicates ubiquitination, and the anti-FLAG band represents *Tg*OTU7. β-Tubulin served as a loading control. The concentrations of the pRK5-*TgOTU7*-FLAG plasmid increased from left to right, as indicated. These antibodies were also used in Panels **C** and **D**. **C** The critical catalytic domain of *TgOTU7* is located between amino acids 149 and 285 (aa). DNA fragments encoding three different regions (1–142 aa, 143–347 aa, 149–285 aa) of *Tg*OTU7 were individually inserted into the pRK5 plasmid to generate mutant *Tg*OTU7 plasmids, which were cotransfected into 293 T cells along with the pRK5-HA-Ub plasmid as labelled. **D** *Tg*OTU7 shows no linkage specificity in its deubiquitinating activity. A fixed amount of recombinant *Tg*OTU7 plasmid was cotransfected into 293 T cells with plasmids containing different lysine-linked Ub chains (K6, K11, K27, K29, K33, K48, and K63). The first lane in each pair contained the linkage-specific substrate as a control, while the second lane contained recombinant *Tg*OTU7 that exhibited deubiquitinating activity, if present.

After the localization at the apicoplast was clearly revealed, we subsequently investigated the function of *Tg*OTU7. First, by aligning the OTU domain of *Tg*OTU7 with *Hs*OTUs, we found that the essential amino acid residues of the catalytic triad, i.e., aspartate [D], cysteine [C], and histidine [H], were largely conserved (Figure 5A). To investigate its deubiquitinase enzymatic activity, we cotransfected HEK293T cells with various amounts of the FLAG-tagged *Tg*OTU7 plasmid and the plasmid pRK5-HA-Ub, followed by Western blotting with anti-HA and anti-FLAG antibodies to determine its ubiquitinating activity and *Tg*OTU7 expression, respectively. Notably, deubiquitinating activity is the reverse action of ubiquitinating activity. The higher the level of deubiquitinating activity is, the lower the level of ubiquitinating activity. As shown in Figure 5B, the deubiquitinating activity of *Tg*OTU7 was concentration dependent, i.e., the greater the level of *Tg*OTU7 was, the greater the deubiquitinating activity.

We next determined the functional domain of the OTUs in *Tg*OTU7. Various DNA fragments, i.e., aa 1–142, aa 143–347 or aa 149–285, were individually cloned and inserted into the pRK5 plasmid. The resultant plasmids were subsequently transfected into HEK293T cells along with pRK5-HA-Ub via the same strategy as that described above. As shown in Figure 5C, the truncated protein spanning aa 143–347 exhibited deubiquitinating activity. In contrast, the protein spanning aa 1–142 did not. The deubiquitinating activity of the resulting product was further mapped to aa 149–285, as predicted (Figure 5C).

Seven lysine (Lys) residues and the N-terminal methionine (Met) of ubiquitin can be ubiquitinated to generate eight linkage types of ubiquitin chains (Met1, Lys6, Lys11, Lys27, Lys29, Lys33, Lys48, and Lys63) [16, 19]. Most mammalian OTUs strongly prefer to cleave polyubiquitin chains with specific linkage types [54, 55]. To examine the ubiquitin linkage-specific activity of *Tg*OTU7, we cotransfected HEK293T cells with the pRK5-*TgOTU7-*FLAG plasmid and one of the seven plasmids containing individual Lys types of ubiquitin chains labelled with an HA tag. Moreover, all seven lysine-linked ubiquitin chains could be trimmed in TgOTU7, indicating no preference for any type of linkage (Figure 5D). Taken together, these results clearly show that the deubiquitinating activity of *Tg*OTU7 is dependent upon the presence of an OTU domain between aa 149 and 285 without linkage specificity.

### *Tg*OTU7 participates in host cell invasion and replication

**Figure 6:**
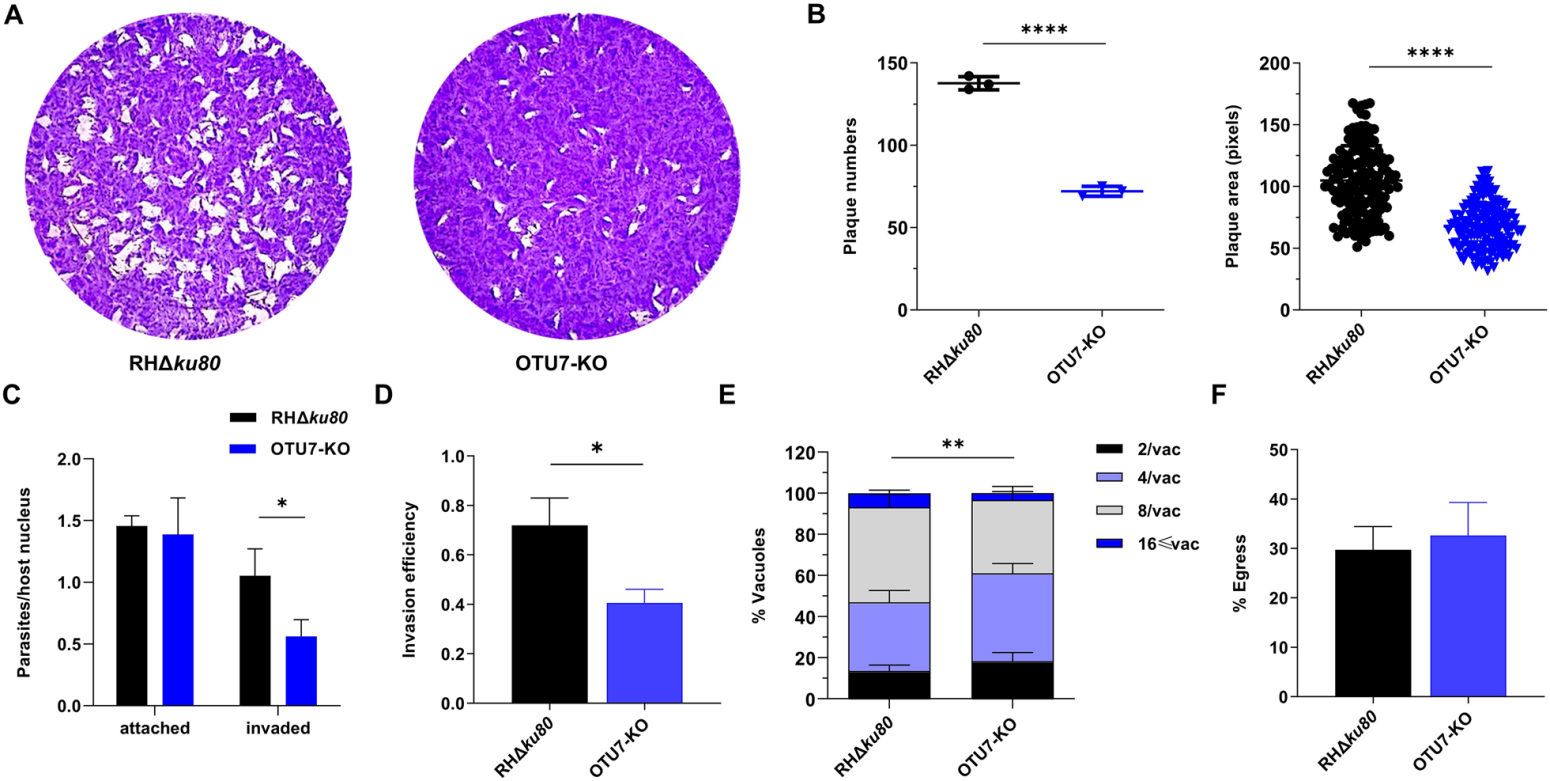
***Tg*OTU7-KO tachyzoites exhibit defects in plaque formation, host cell invasion and replication.** **A** Plaques formed from HFFs by the RHΔ*ku80* and *Tg*OTU7-KO parasites. Plaques are shown as clear spots on the violet background of a monolayer of HFFs stained with crystal violet. **B** Quantification of the plaque number (left) and area (right) of the RHΔ*ku80* and *Tg*OTU7-KO parasites. **C** Attachment and invasion of RHΔ*ku80* and *Tg*OTU7-KO parasites were quantified as the ratio of attached or invaded parasites to the host nucleus. **D** Quantification of the invasion efficiency of RHΔ*ku80* and *Tg*OTU7-KO parasites, which was quantified as the ratio of invaded parasites to total parasites. **E** Replication of RHΔ*ku80* and *Tg*OTU7-KO parasites. The quantification of parasites per parasitophorous vacuole is shown. **F** Induced egress of RHΔ*ku80* and *Tg*OTU7-KO parasites. All the data were collected from three independent experiments. Statistical analysis was performed with an unpaired t test, *P* values: * ≤ 0.05, ** ≤ 0.01, **** ≤ 0.0001.

Next, we genetically dissected the role of *Tg*OTU7 in the lytic cycle biology of *Toxoplasma*. A *Tg*OTU7 knockout (*Tg*OTU7-KO) and a knockout complemented strain (*Tg*OTU7-COM) were generated using the strategy detailed in the Methods section and illustrated in Additional file 4, and PCR and DNA sequencing were used for confirmation. The growth of *Tg*OTU7-KO mice was monitored by the formation of plaques (Figure 6A), which is a reliable in vitro method for quantifying tachyzoite lytic capacity [33]. Similarly, compared with those of the parental RHΔ*ku*80 plants, the number and area of the plaques formed by the *Tg*OTU7-KO plants were 65% and 37%, respectively (Figure 6B,  *P* < 0.0001). The defects in plaque formation were restored in the number and area of *Tg*OTU7-COM plants (Additional file 5). These data showed that the loss of *Tg*OTU7 function affects the lytic cycle in vitro. The lytic cycle is characterized by repeated rounds of motility, host cell adhesion, invasion, replication, and egress [56]. To determine which gene was impacted in the *Tg*OTU7-KO mice, standard assays were individually performed. The invasion rate, defined as the ratio of invaded parasites/host nuclei of the *Tg*OTU7-KO mice, was 46.6% lower than that of the RHΔ*ku80* mice (Figure 6C,   *P* < 0.05). Similarly, the invasion efficiency of the knockout strain, which represented invaded parasites/total parasites, was 43.6% lower (Figure 6D,   *P* < 0.05). However, the attachment of *Tg*OTU7-KO mice, presented as the ratio of attached parasites to host nuclei, was not affected (Figure 6C,   *P* > 0.05). At 24 h post-infection, *Tg*OTU7-KO mice had fewer tachyzoites per vacuole than did the RHΔ*ku80* mice, as determined by standard doubling assays (Figure 6E, *P* < 0.01). Finally, no significant difference in egress ability from host cells was observed between the *Tg*OTU7-KO and wild-type RHΔ*ku80* strains (Figure 6F). Collectively, our data show that *Tg*OTU7 plays a role in host cell invasion and replication during the lytic cycle.

### *Tg*OTU7 affects apicoplast biogenesis and transcription

**Figure 7:**
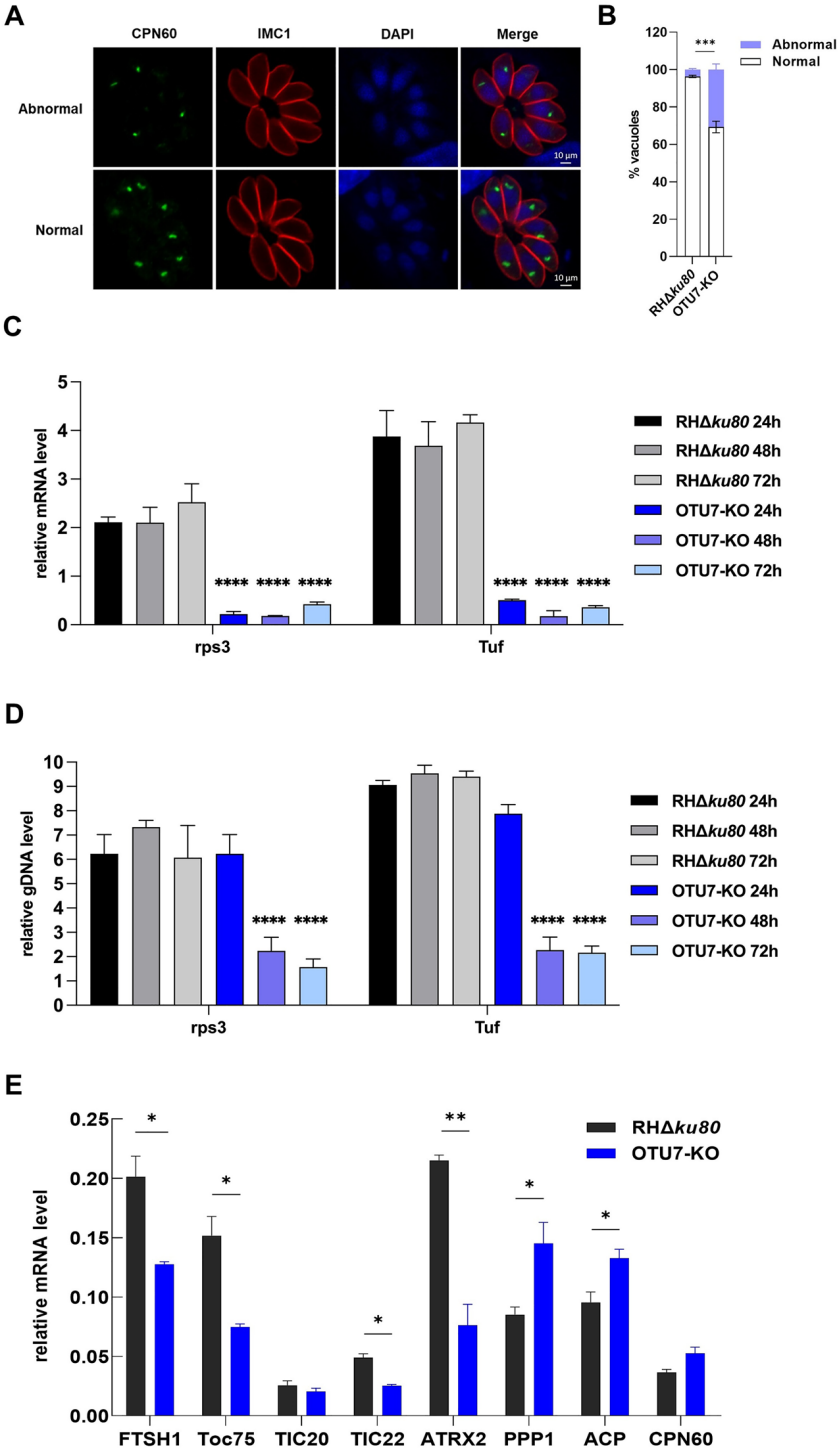
**Loss of function of *Tg*OTU7 affects** ***Toxoplasma gondii*** **biogenesis and genomic transcription of the apicoplast.** **A** Abnormal apicoplast in *Tg*OTU7-KO mice. Parasites were stained with rabbit anti-CPN60 (green), mouse anti-IMC1 (red) and DAPI (blue) for immunofluorescence. Some daughter cells of *Tg*OTU7-KO plants lost apicoplasts during division. **B** Quantification of abnormal apicoplasts in *Tg*OTU7-KO mice, as shown in Panel **A**. **C** and **D** Quantification of mRNA (**C**) and genomic DNA (**D**) via qRT‒PCR are shown for the rps3 and Tuf genes. The data are from three independent experiments performed in triplicate. **E** Relative mRNA levels of the nuclear genes whose protein products targeted the apicoplast were quantified by qRT‒PCR. Statistical analysis was performed using the 2^−ΔΔCT^ method in Excel and two-way ANOVA or unpaired t test. *P* values: * ≤ 0.05, ** ≤ 0.01, *** ≤ 0.001, **** ≤ 0.0001.

The roles of *Tg*OTU7 in apicoplast biogenesis were investigated next. The process of apicoplast division and partitioning is essential during each cellular cycle to ensure the equal distribution of organelles to progeny cells. Consequently, apicoplast division is tightly regulated in coordination with the progression of the cell cycle [57]. Many daughter cells of *Tg*OTU7-KO plants were found to have lost apicoplasts during division, indicating an abnormality in apicoplast biogenesis. Specifically, approximately 31% of apicoplasts were abnormal in *Tg*OTU7-KO mice, whereas only 4% were abnormal in the parental control according to IFA using CPN60, a lumen protein marker (Figures 7A and B). This abnormality in *Tg*OTU7-KO mice was apicoplast specific since the Golgi apparatus, rhoptries and dense granules all appeared normal according to IFA using specific markers of each organelle (Additional file 6).

There are 30 genes in the apicoplast genome, which mostly encode ribosomal proteins and elongation factors [58]. To determine the role of *Tg*OTU7 in apicoplast genomic transcription, two representative genes, ribosomal protein S3 (rps3) and elongation factor tu (Tuf), were quantified via qRT‒PCR. Total RNA and gDNA were extracted from *Tg*OTU7-KO mice harvested after 24 h, 48 h, and 72 h of culture. Compared to those in the controls, 89.7% and 86.9% decreases in the rps3 and Tuf transcripts, respectively, were observed beginning at 24 h. In contrast, a decrease in the abundance of apicoplast gDNA occurred later, which was caused by abnormalities in apicoplast biogenesis (Figures 7C and D). These results unequivocally showed that the decrease in the mRNA levels of apicoplast-encoded genes was not solely due to apicoplast loss and suggested that this decrease in *Tg*OTU7-KO was also due to an impact on apicoplast genome transcription.

Furthermore, since most apicoplast proteins are encoded by the nuclear genome and imported into the organelle, we selected several genes that encode proteins localized in different regions of the apicoplast and assessed their transcripts (Additional file 7). Among the eight genes tested, ATRX2 and PPP1 exhibited the greatest decrease and increase in transcription, i.e., 64% and 70%, respectively (Figure 7E). Nevertheless, a definitive conclusion cannot be drawn about the role of TgOTU7 in apicoplast protein import based on these data since *Tg*OTU7, a DUB, is more likely to influence protein function at the post-translational level. Collectively, our data showed that *Tg*OTU7 plays a pivotal role in apicoplast biogenesis and transcription.

## Discussion

*Toxoplasma gondii* possesses a complete system of protein ubiquitination, which has also been found in several other organisms [53, 59, 60]. Ubiquitination plays crucial roles in vital physiological processes of *T. gondii*, such as cell cycle progression and IMC biogenesis [53]. In addition to the cytoplasm and nucleus, ubiquitination occurs in the apicoplast, a unique organelle of apicomplexan protozoa. The latter may be equipped with the necessary components needed for ubiquitination [25, 26]. Little is known about deubiquitases, important components of the ubiquitination system, in *T. gondii*.

In this study, we focused on *Toxoplasma* OTUs, especially those of *Tg*OTU7. The OTU family of DUBs has recently attracted increased amounts of attention. The first identified OTU was the ovarian tumour gene of *Drosophila melanogaster* [61], followed by the discovery of 16 *Hs*OTUs in the human genome [62]. *T. gondii* has many diverse OTUs [55, 60], for which at least 12 were predicted. One of them*, Tg*OTUB1, is predicted to localize to the apicoplast [22]. Our first task was to confirm this prediction, which was inaccurate since *Tg*OTUB1 was not found to be localized at the apicoplast (Figure 1). We then addressed whether the remaining 11 OTUs were localized at the apicoplast. We have tagged most DUBs in *Toxoplasma* (unpublished). Among all the DUBs tested, only *Tg*OTU7 was found to be localized at the apicoplast (Figure 4), representing the first deubiquitinase of *T. gondii* OTUs with a confirmed location at the apicoplast.

Most *Hs*OTUs clearly preferred to cleave polyubiquitin chains with specific linkages. For instance, *Hs*OTUD1 favours the cleavage of K48-linked chains [54]. We have convincingly shown here that the deubiquitination activity of *Tg*OTU7 occurs on all seven possible Lys-linked chains with no specificity, undoubtedly demonstrating that *Tg*OTU7 is functionally different from *Hs*OTUs. These findings also support the notion that *Tg*OTU7 is the sole deubiquitinase localized at the apicoplast of *T. gondii* and is responsible for cleaving various ubiquitinated proteins in the organelle. The linkage specificity of OTUs is related to their ubiquitin-binding domains [54]. While we have identified the OTU domain of *Tg*OTU7, further structural studies are needed to explore its nonspecific deubiquitinating activity. Recently, K11-linked chains have emerged as important regulators of the ERAD system [63]. Furthermore, several lines of evidence show an interaction between OTU1 of different species and components of the ERAD pathway [64–66]. *Tg*OTU7 might be a major player in the ERAD system of *T. gondii* due to its deubiquitinating activity in the apicoplast. Nonetheless, all the deubiquitinating activity experiments performed in the present study were conducted in the 293 T cell line, and in vivo experiments involving infection with *T. gondii* are needed. OTU deubiquitinases are involved in various cellular processes, such as DNA damage repair, NF-kB signalling and the immune response [67–69]. Moreover, *Hs*OTUs play roles in cell cycle progression by participating in cell proliferation and differentiation [67, 70]. Here, we showed that the expression of *Tg*OTU7 is intricately regulated during the parasite’s cell cycle, consistent with the observation that a subset of ubiquitin ligases and deubiquitinases are regulated by the cell cycle [53]. A range of ubiquitinated proteins have been found to localize to the apicoplast, and two peaks of protein ubiquitination are observed each in the G1 and S/M phases [53], which corresponds to the expressional peak of *Tg*OTU7. It is very likely that *Tg*OTU7 is involved in the complex ubiquitome of *T. gondii* and thus regulates cell cycle progression. Furthermore, by knocking out *Tg*OTU7, we showed that *Tg*OTU7 contributes to cell growth, replication and invasion. It also plays roles in apicoplast homeostasis and inheritance. Given that *Tg*OTU7 is expressed throughout the entire cell cycle, with a peak in the G1/S phase, it is not unexpected that the absence of *Tg*OTU7 causes moderate defects in parasite replication. Currently, why *Tg*OTU7 KO leads to a decrease in *Toxoplasma* invasion efficiency is unclear and possibly results from the involvement of pathways related to the apicoplast in host-cell invasion and infection [9, 71]. From a subcellular perspective, the loss of apicoplasts in *Tg*OTU7-KO mice may also contribute to growth inhibition, considering the importance of coordinating organelle division and the cell cycle. Our findings also revealed that transcription of the apicoplast genome was downregulated in the *Tg*OTU7-KO zebrafish, which was partially due to the loss of the apicoplast and possibly other unelucidated biological defects.

By evaluating the transcriptional levels of the nuclear genes encoding the proteins imported to the apicoplast, we found that *Tg*OTU7 possibly participated in the ERAD system. Most proteins inside the second outermost membrane had a higher transcriptional level in the *Tg*OTU7-KO zebrafish than in the parental control, whereas those outside the membrane had a low level of transcription. The ERAD system essentially functions in protein import through the second innermost membrane of the apicoplast [28]. Therefore, it is plausible that the loss of *Tg*OTU7 results in defects in protein translocation to the apicoplast and that the parasites compensate for this change by increasing the expression of relevant proteins. The apicoplast ERAD system retains the ability to ubiquitinate proteins, which play an important role in protein import into the apicoplast [25, 26]. The presence of a ubiquitin-like protein in the apicoplast implies that an apicoplast-specific deubiquitinase is needed for protein translocation [26]. Recent studies have shown that Otu1 in yeast [64], OTUD2 in humans [72], and OTU1 in Arabidopsis [73] are important components associated with Cdc48 in the ERAD system. Moreover, two distinct Cdc48 proteins have been found in *T. gondii*, one of which is localized to the periplastid compartment (PPC) of the apicoplast [48]. As the only deubiquitinase discovered thus far to be located in the apicoplast, *Tg*OTU7 represents a key component of the *T. gondii* ERAD system. Nevertheless, we have not addressed the roles that *Tg*OTU7 plays in protein import into the apicoplast. *Tg*OTU7 may be involved in the post-transcriptional modification of these proteins, which remains to be confirmed.

The apicoplast of Apicomplexan protozoa is a potential target for chemical treatment due to its evolutionary origin from algae. This origin implies that the organelle contains unique proteins and pathways that are distinct from those in mammalian cells [10]. In fact, *Tg*OTU7 is phylogenetically divergent from *Hs*OTUs, and drugs targeting *Tg*OTU7 are likely to have minimal toxic effects on its mammalian host. Therefore, as the only apicoplast DUB, *Tg*OTU7 is not only an entity for decoding the ubiquitin code of *T. gondii* but also a promising target for the development of new drugs against this ubiquitous pathogenic parasite.

### Supplementary Information

**Additional file 1. Phylogenetic tree of** ***Tg*OTU7 and 15 human OTUs.** A phylogenetic tree was constructed based on the amino acid sequences with the maximum likelihood algorithm using MEGA X. Bootstrap analysis was performed with 100 replicates. The scale bar indicates the evolutionary distance between the sequences.

**Additional file 2. Sequence similarity of** ***Tg*OTU7 homologues among different Apicomplexa species.**

**Additional file 3. Robust multiarray average (RMA)-normalized signal intensity values of the** ***TgOTU7*** **gene**. The statistical analysis was performed as previously described [74]. The expression levels of CPN60 and *Tg*OTU7 are synchronized during the life cycle of *Toxoplasma gondii*. The change in *Tg*OTU7 expression depended on the cytokinesis stage, with the highest level occurring in the early stage and the lowest occurring in the late stage.

**Additional file 4. Construction and identification of the** ***Tg*OTU7 knockout strain.** A. Schematic representation of the CRISPR/Cas9 strategy used to construct *Tg*OTU7-KO tachyzoites. B. PCR analysis of the *Tg*OTU7 gene knockout. PCR targets are depicted in Panel A. C. Schematic diagram of the CRISPR/Cas9 strategy used to construct *Tg*OTU7-COM tachyzoites. D. PCR analysis of the *Tg*OTU7 gene complement. PCR targets are depicted in Panel C.

**Additional file 5. The** ***Tg*OTU7-COM strain restored plaque formation.** A. Plaques formed by the RHΔ*ku80*, *Tg*OTU7-KO and *Tg*OTU7-COM parasites in HFF cells. B. Quantification of the plaque number (left) and area (right) of the RHΔ*ku80*, *Tg*OTU7-KO and *Tg*OTU7-COM parasites. Two-way ANOVA was used for statistical analysis, *P* values: **** ≤ 0.0001.

**Additional file 6. Immunofluorescence analysis of the influence of** ***Tg*OTU7 on cellular organelles.** Loss of *Tg*OTU7 did not visibly change the Golgi apparatus, rhoptries or dense granules. Parasites were stained with mouse anti-Sortilin (green), mouse anti-GRA16 (green), mouse anti-ROP14 (green), rabbit anti-IMC1 (red) and DAPI (blue).

**Additional file 7. Locations of the 8 confirmed nuclear-encoded apicoplast genes selected for qRT‒PCR.**

## Data Availability

All the data generated or analysed during this study are included in this published article and its supplementary information files.
