# Supplementary figures and images for: An apicoplast-localized deubiquitinase contributes to the cell growth and apicoplast homeostasis of Toxoplasma gondii
Source: Vet Res. 2024 Jan 17;55:10. doi: 10.1186/s13567-023-01261-y (PMC10795397; doi:10.1186/s13567-023-01261-y)

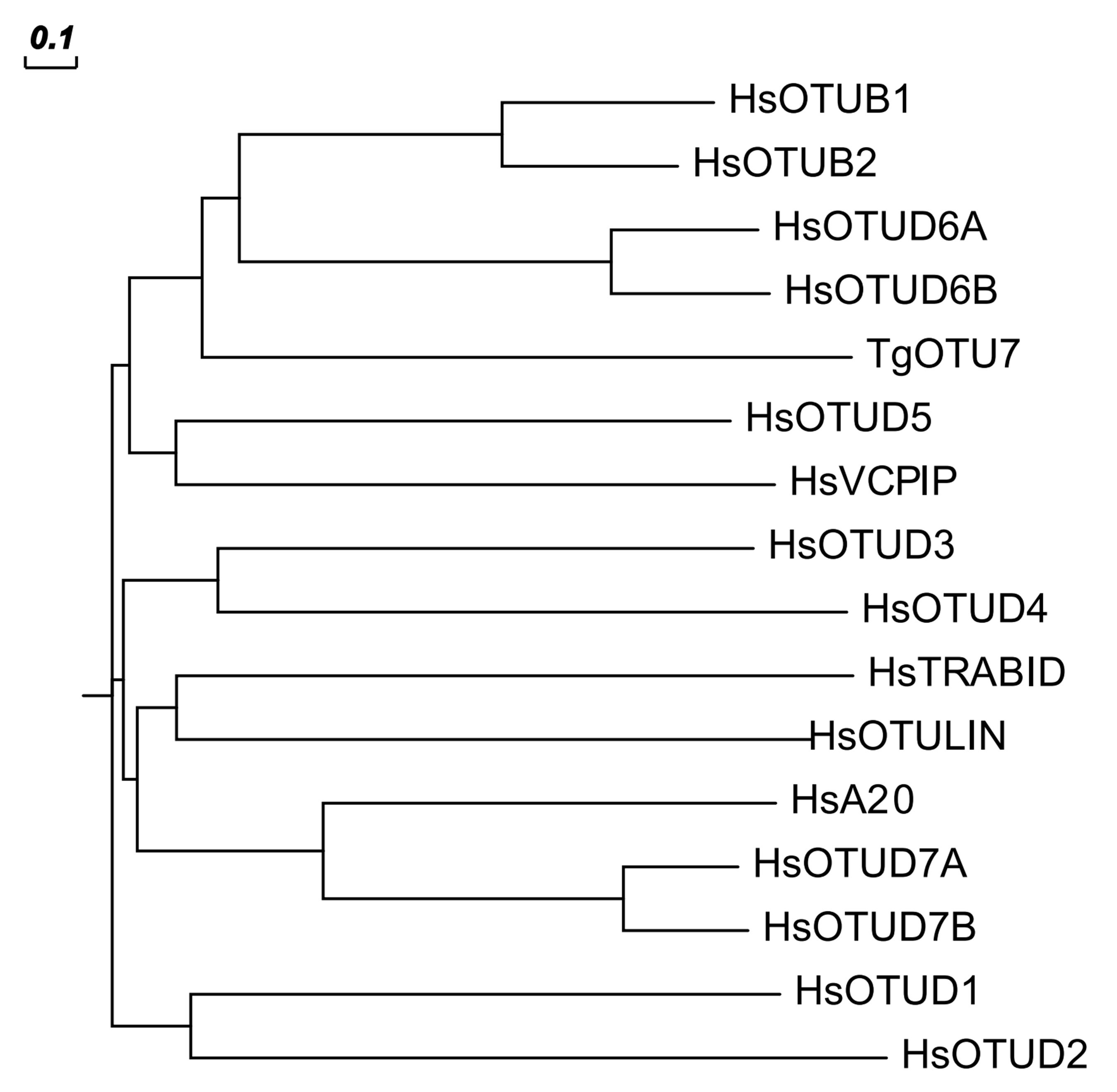

Supplement: Supplementary file 1 — Additional file 1. Phylogenetic tree of TgOTU7 and 15 human OTUs. A phylogenetic tree was constructed based on the amino acid sequences with the maximum likelihood algorithm using MEGA X. Bootstrap analysis was performed with 100 replicates. The scale bar indicates the evolutionary distance between the sequences. [file 13567_2023_1261_MOESM1_ESM.jpg]

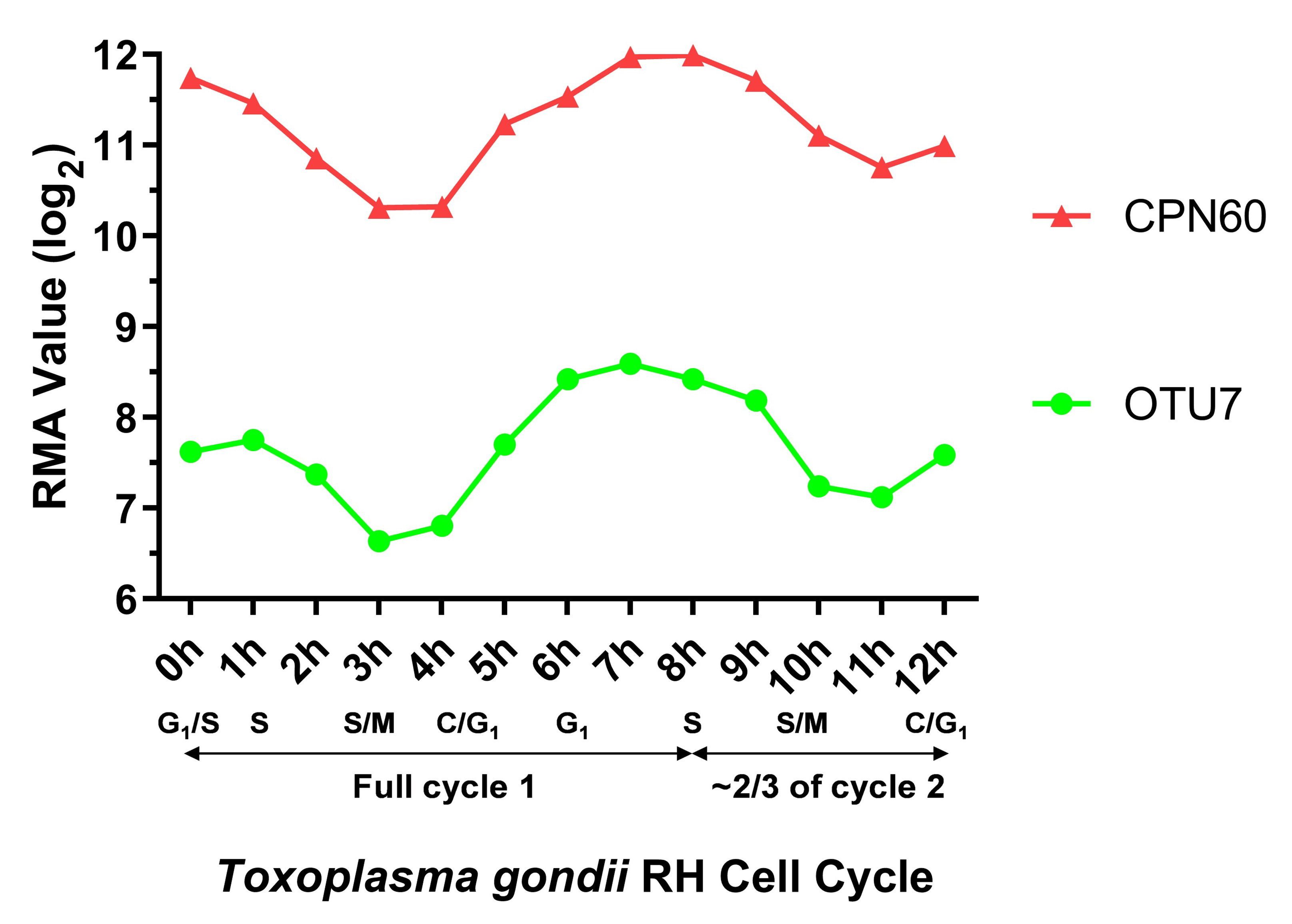

Supplement: Supplementary file 3 — Additional file 3. Robust multiarray average (RMA)-normalized signal intensity values of the TgOTU7 gene. The statistical analysis was performed as previously described [74]. The expression levels of CPN60 and TgOTU7 are synchronized during the life cycle of Toxoplasma gondii. The change in TgOTU7 expression depended on the cytokinesis stage, with the highest level occurring in the early stage and the lowest occurring in the late stage. [file 13567_2023_1261_MOESM3_ESM.jpg]

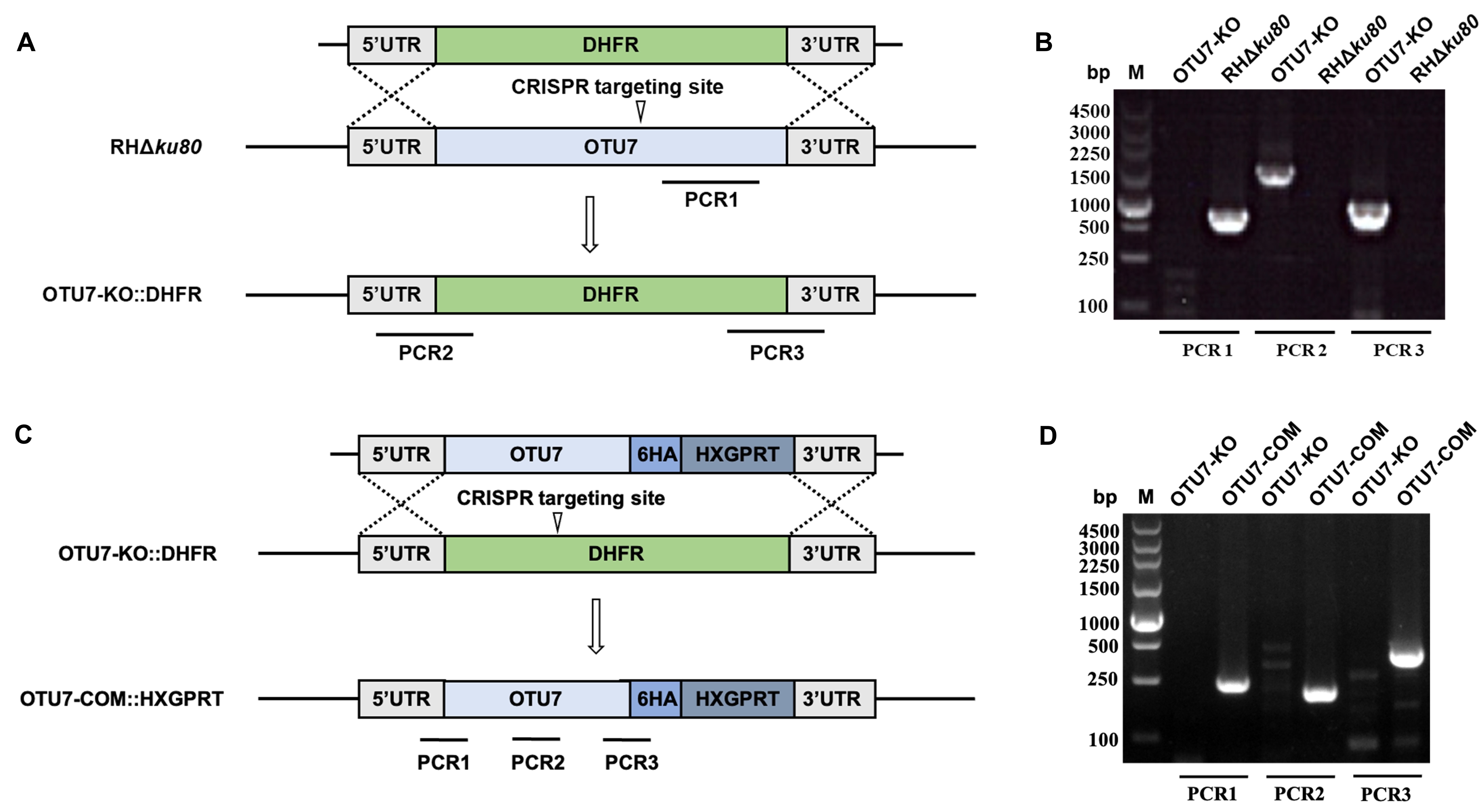

Supplement: Supplementary file 4 — Additional file 4. Construction and identification of the TgOTU7 knockout strain. A. Schematic representation of the CRISPR/Cas9 strategy used to construct TgOTU7-KO tachyzoites. B. PCR analysis of the TgOTU7 gene knockout. PCR targets are depicted in Panel A. C. Schematic diagram of the CRISPR/Cas9 strategy used to construct TgOTU7-COM tachyzoites. D. PCR analysis of the TgOTU7 gene complement. PCR targets are depicted in Panel C. [file 13567_2023_1261_MOESM4_ESM.jpg]

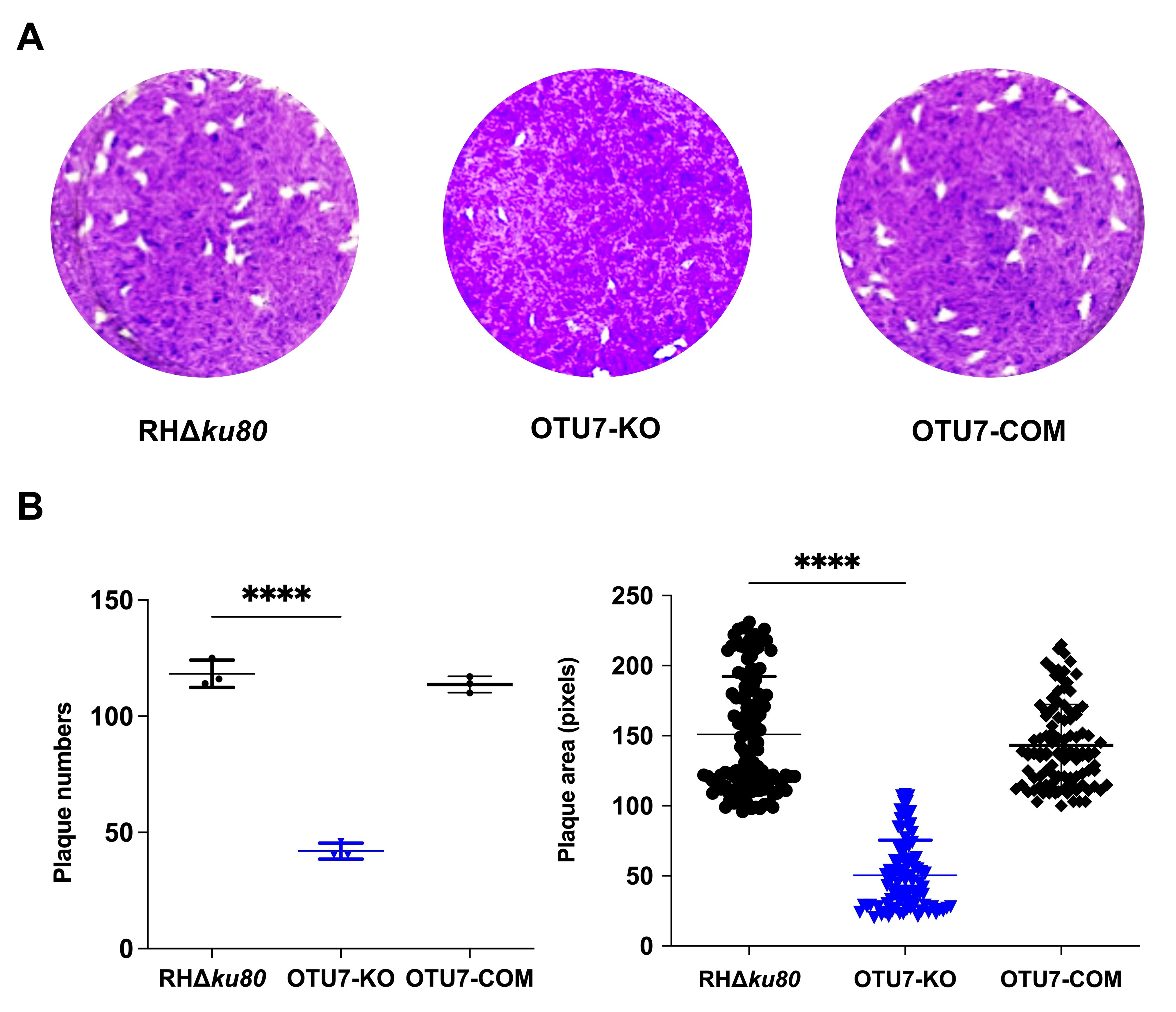

Supplement: Supplementary file 5 — Additional file 5. The TgOTU7-COM strain restored plaque formation. A. Plaques formed by the RHΔku80, TgOTU7-KO and TgOTU7-COM parasites in HFF cells. B. Quantification of the plaque number (left) and area (right) of the RHΔku80, TgOTU7-KO and TgOTU7-COM parasites. Two-way ANOVA was used for statistical analysis, P values: **** ≤ 0.0001. [file 13567_2023_1261_MOESM5_ESM.jpg]

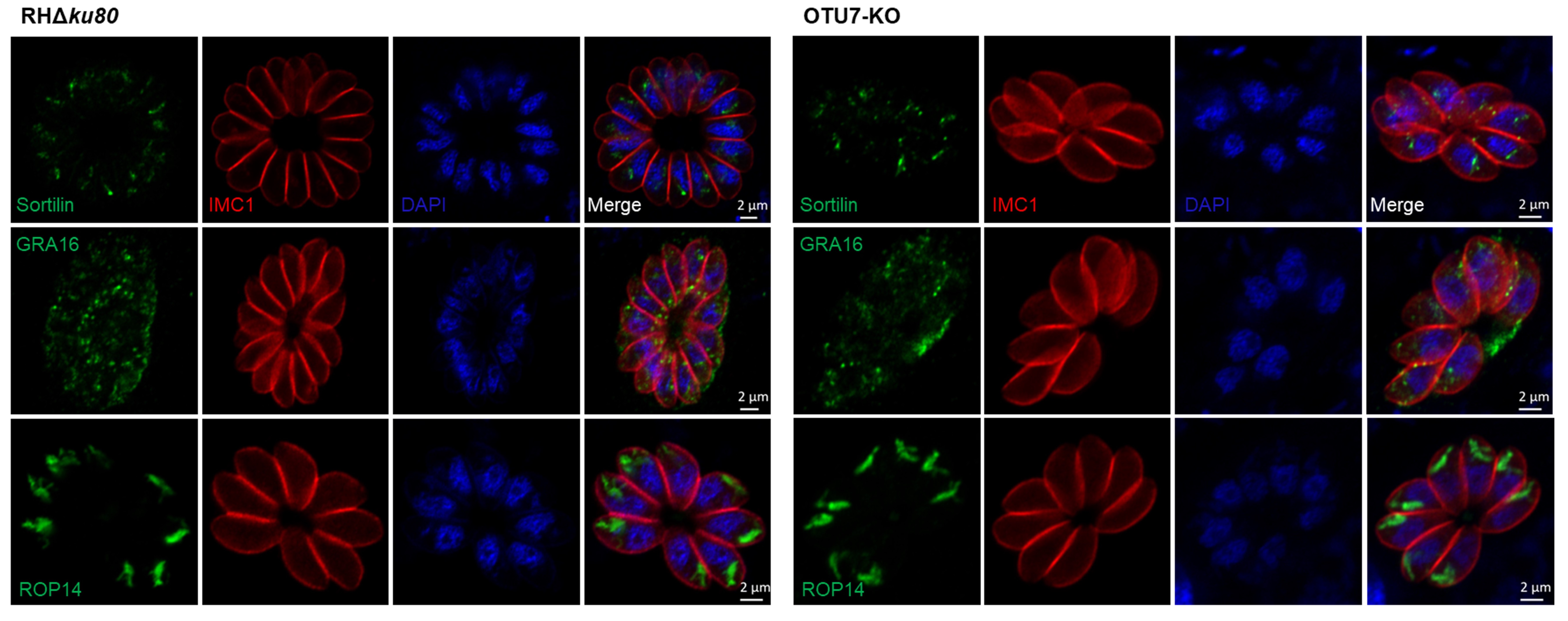

Supplement: Supplementary file 6 — Additional file 6. Immunofluorescence analysis of the influence of TgOTU7 on cellular organelles. Loss of TgOTU7 did not visibly change the Golgi apparatus, rhoptries or dense granules. Parasites were stained with mouse anti-Sortilin (green), mouse anti-GRA16 (green), mouse anti-ROP14 (green), rabbit anti-IMC1 (red) and DAPI (blue). [file 13567_2023_1261_MOESM6_ESM.jpg]
