# Supplementary material for: An apicoplast-localized deubiquitinase contributes to the cell growth and apicoplast homeostasis of Toxoplasma gondii
Source: Vet Res. 2024 Jan 17;55:10. doi: 10.1186/s13567-023-01261-y (PMC10795397; doi:10.1186/s13567-023-01261-y)
Supplement: Supplementary file 7 — Additional file 7. Locations of the 8 confirmed nuclear-encoded apicoplast genes selected for qRT‒PCR. [file 13567_2023_1261_MOESM7_ESM.docx]

**Additional file 7. Localization of 8 confirmed nuclear-encoded apicoplast genes selected for qRT-PCR.**

| Name | GeneID | Localization |
| --- | --- | --- |
| FTSH1 | TGGT1_259260 | Outermost |
|  |  |  |
| Toc75 | TGGT1_272390 | Second inner-most |
|  |  |  |
| TIC20 | TGGT1_255370 | Innermost |
|  |  |  |
| TIC22 | TGGT1_286050 | Innermost |
|  |  |  |
| ATRX2 | TGGT1_310770 | PPC^a^ |
|  |  |  |
| PPP1 | TGGT1_287270 | PPC |
|  |  |  |
| ACP | TGGT1_264080 | Luminal |
|  |  |  |
| CPN60 | TGGT1_240600 | Luminal |

^a^PPC: periphery compartment
